# Why Is the Prevention of Passive Transport of Cat Allergens So Important in Clinical Practice?

**DOI:** 10.1002/clt2.70197

**Published:** 2026-09-02

**Authors:** Gennaro Liccardi, Matteo Martini, Maria Beatrice Bilò, Lorenzo Cecchi, Manlio Milanese, Roberta Maniscalco, Paola Rogliani

**Affiliations:** ^1^ Postgraduate School of Respiratory Medicine, Department of Experimental Medicine University of Rome “Tor Vergata” Rome Italy; ^2^ Allergy Unit, Department of Internal Medicine University Hospital A.O.U. delle Marche Ancona Italy; ^3^ Department of Clinical and Molecular Sciences Marche Polytechnic University Ancona Italy; ^4^ Allergy and Immunology Unit San Giovanni di Dio Hospital, USL Toscana Centro Florence Italy; ^5^ Division of Pulmonology S. Corona Hospital Pietra Ligure Italy; ^6^ Department of Veterinary University of Naples Federico II Naples Italy; ^7^ Department of Experimental Medicine, Unit of Respiratory Medicine University of Rome “Tor Vergata” Rome Italy

**Keywords:** cat, cat and dog allergens, clothing, dog, indirect exposure, passive transport, prevention

To the Editor

We read with interest the article of Colosimo et al. [1] concerning innovative strategies to reduce exposure and consequent allergic sensitization to cat allergen Fel d 1.

The article is very complete, and we have no objections to its contents. However, we wish to address an additional and universal exposure pathway that is the passive transport of cat allergens in animal‐free environments and, above all, the lack of information on preventive measures to this crucial aspect.

This clarification is important because the majority of the innovative strategies reported in the text are applicable just to the single cat that is owned by the patient, or to the indoor environment where the animal lives. Obviously, patients remain exposed to the allergens/environments of other cats that they may encounter. On the contrary, targeted prevention measures against passive transport have a universal character as they should reduce the phenomenon of the “ubiquity” of the cat allergen and therefore the risk of sensitization without contact with animals.

Cat (and dog) allergens should be considered as ubiquitous because they are found not only in indoor environments where these animals are kept, but also in other indoor private or public places where they have never been kept. Public spaces include nurseries, offices, hospitals, hotels, schools and means of public transport. These indoor environments, contaminated by cat allergens, are able to induce allergic sensitization in susceptible individuals and trigger respiratory symptoms in already highly sensitized subjects. In fact, in these contaminated environments, the amount of cat allergens is higher than clinically significant thresholds. Namely, 1 and 8–10 μg of allergen/g are generally recognized as sufficient to induce sensitization or trigger respiratory symptoms for dust, respectively. Obviously, these thresholds must be considered as “averages” over the population examined in clinical studies. However, in clinical practice, it is necessary to account the wide individual susceptibility to pet allergen exposure resulting from genetic and environmental factors, as these variables are able of influencing the onset of more or less severe symptoms.

In developed countries, the cat allergen ubiquity causes a persistent stimulation of airways like that induced by dust mite.

Accumulation of cat allergens in indoor environments without animals is correlated with the number of visitors owning a cat or with those who are in regular contact with these animals. We have shown that clothing [2], but also human hair [3], constitute a means of transferring cat allergens into pet‐free indoor environments (Figure 1).

**FIGURE 1:**
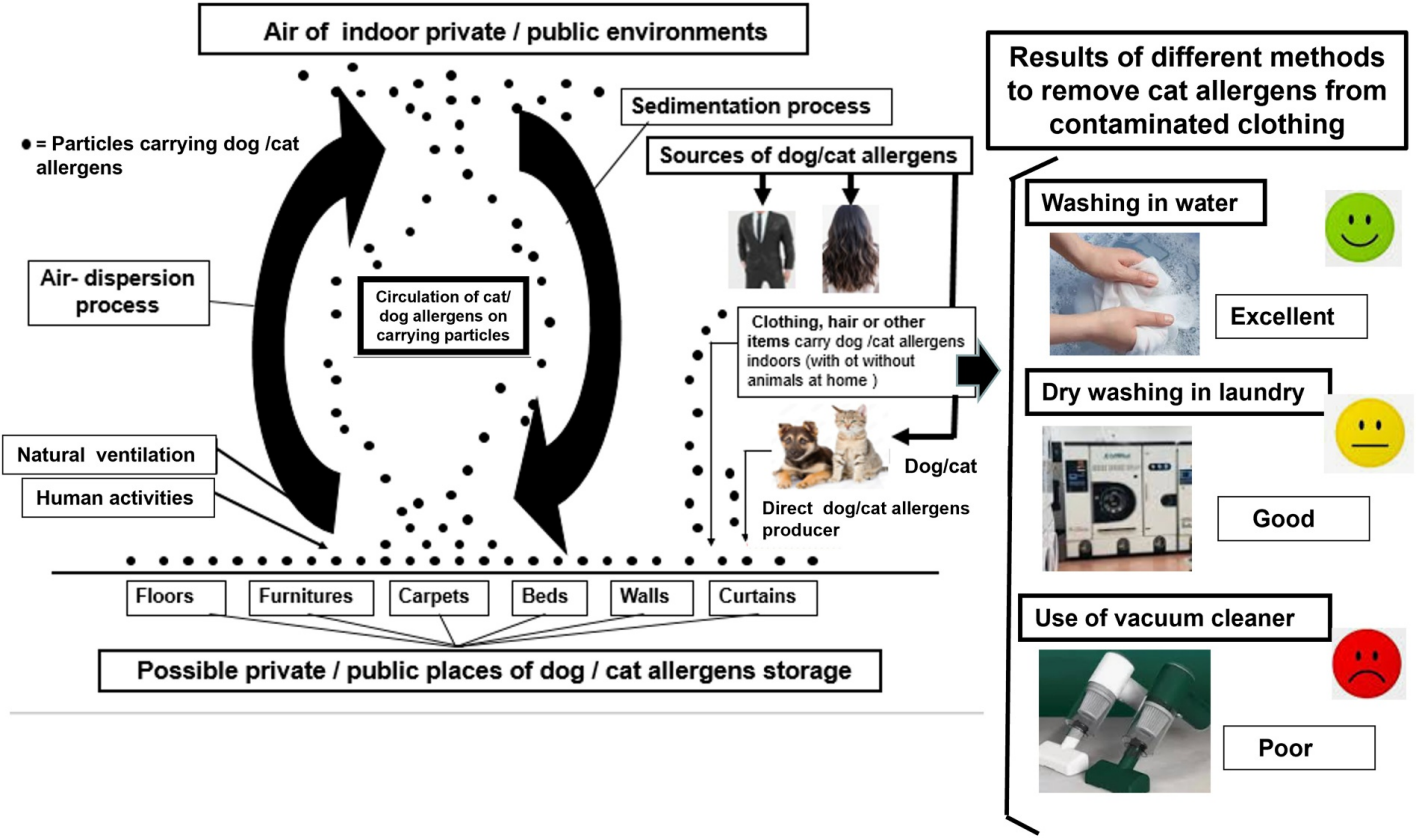
Dynamics of cat/dog allergens in indoor private/public environments and possible means to remove cat allergens from contaminated clothing. Modified from ref. [4].

We have previously demonstrated it in a less common pet (rabbit) [5]. However, a smaller body of evidence suggests the possibility of passive transport of allergens also in other animals such as horses [6, 7], rodents [8] and cattle [9]. For these latter animals, the most common cause of passive transport is occupational. Indeed, breeders, farmers, animal handlers, and other professionals can transfer these animals' allergens from their workplaces to their homes, presumably through clothing or other items.

Therefore, we believe it is important to mention the validity of different cleaning methods in removing cat allergens from passively contaminated wool fabrics. We have previously demonstrated the efficacy of washing these fabrics in water (excellent) [6, 7, 8, 9, 10] compared to dry cleaning (good) [7, 8, 9, 10, 11] and vacuuming (poor) [8, 9, 10, 11, 12] (Figure 1).

The need to wear clothes that carry minimal amounts of cat/dog allergens becomes crucial to avoid their indoor accumulation in some places such as hospitals, clinics, nurseries, etc. An example could be the entry of dogs and trainers into pediatric hospital departments for pet therapy. In Sweden, for entry certification, washing the hospital dog before entering is required. We proposed including in these Guidelines some precautions regarding the clothing of hospital dog owners/trainers [13].

As a priority, we suggested changing private clothes and using hospital clothing and a disposable cap for hair. Alternatively, it may be useful to wear disposable gowns over clothes, always with head covered by a disposable cap. We suggest the use of these materials even by ordinary visitors who have constant/direct contact with pets [9, 10, 11, 12, 13].

In conclusion, we believe that preventing the passive spread of cat allergens is an important measure to try to reduce indoor contamination and consequent allergic sensitization. Further “innovative” studies should be planned also on this topic in the future, such as evaluating the effectiveness of proteolytic enzymes (e.g. Subtilisin A) applied to patients' clothes, that could be easier than repeated washing. Unfortunately, these procedures are still in the preliminary laboratory stage, and further studies are needed to confirm their effectiveness in real‐world conditions and, above all, to minimize possible adverse effects on airways and skin.

## Author Contributions

**Gennaro Liccardi:** conceptualization, writing – original draft, supervision. **Matteo Martini:** writing – review and editing, supervision. **Maria Beatrice Bilò:** writing – review and editing. **Lorenzo Cecchi:** writing – review and editing. **Manlio Milanese:** writing – review and editing. **Roberta Maniscalco:** writing – review and editing. **Paola Rogliani:** writing – review and editing.

## Funding

The authors have nothing to report.

## Conflicts of Interest

The authors declare no conflicts of interest.

## Summary at a Glance

The prevention of passive transport of pet (cat/dog) allergens in pet‐free environments appears to be an indispensable measure to reduce the degree of allergic sensitization to pets in susceptible population.

## Data Availability

The data that support the findings of this study are available on request from the corresponding author. The data are not publicly available due to privacy or ethical restrictions.
